# Benefits of a Balance Exercise Assist Robot in the Cardiac Rehabilitation of Older Adults with Cardiovascular Disease: A Preliminary Study

**DOI:** 10.3390/jcdd9060191

**Published:** 2022-06-12

**Authors:** Kakeru Hashimoto, Akihiro Hirashiki, Kenichi Ozaki, Koki Kawamura, Junpei Sugioka, Shunya Tanioku, Kenji Sato, Ikue Ueda, Naoki Itoh, Kenichiro Nomoto, Manabu Kokubo, Atsuya Shimizu, Izumi Kondo

**Affiliations:** 1 Department of Rehabilitation, National Center for Geriatrics and Gerontology, Obu 474-8511, Japan; khashi@ncgg.go.jp (K.H.); ozk-kety@ncgg.go.jp (K.O.); kawamura@ncgg.go.jp (K.K.); sugioka@ncgg.go.jp (J.S.); t-shun8@ncgg.go.jp (S.T.); k-sato@ncgg.go.jp (K.S.); ikueueda@ncgg.go.jp (I.U.); n-itoh@ncgg.go.jp (N.I.); ik7710@ncgg.go.jp (I.K.); 2 Department of Cardiology, National Center for Geriatrics and Gerontology, Obu 474-8511, Japan; yumahiro@ncgg.go.jp (K.N.); mkokubo@ncgg.go.jp (M.K.); ashimizu@ncgg.go.jp (A.S.)

**Keywords:** cardiovascular disease, balance exercise assist robot, older adults, frailty, cardiac rehabilitation, robotic rehabilitation

## Abstract

We examined whether adding robot-supported balance exercises to cardiac rehabilitation improves the ability to balance in older adults with cardiovascular disease (CVD). We conducted a prospective study in 52 older adults who had been hospitalized for worsening CVD. Once weekly for four months, for a total of sixteen sessions as outpatients, the subjects used a Balance Exercise Assist Robot (BEAR) to perform balance exercises and an ergometer for aerobic exercises. Participants’ mean age was 76.9 ± 6.8 years (range, 65–95 years), and their mean brain natriuretic protein level was 164.0 ± 190.0 pg/mL. After the intervention, participants showed significant improvements in gait speed (before, 1.06 ± 0.33 m/s; after, 1.23 ± 0.30 m/s; *p* < 0.001), Short Physical Performance Battery score (before, 10.02 ± 2.25; after, 10.88 ± 1.79; *p* ˂ 0.001), timed up-and-go (before, 11.11 ± 5.07 s; after, 9.45 ± 3.45 s; *p* ˂ 0.001), and knee extension (before, 26.97 ± 11.78 kgf; after, 30.13 ± 13.04 kgf; *p* = 0.001). Cardiac rehabilitation including exercises using BEAR improved physical functioning and the ability to balance in older adults with CVD. Frail and prefrail patients improved, whereas robust ones did not change.

## 1. Introduction

The number of patients with cardiovascular disease (CVD) increases with age [1]. Older adults with CVD often have multiple comorbidities, including reduced exercise capacity, decreased physical and cognitive functions, and depression [2]. In addition, they frequently have problems typical of older adults in general, including frailty [3] and sarcopenia [4]. Frailty encompasses physical, social, and cognitive/depression aspects that contribute to both adverse cardiovascular outcomes and non-cardiovascular mortality [5]. These various problems decrease the life expectancy of older adults with CVD [6,7].

Few data are available regarding balance function in people with CVD [8]. These patients often have skeletal muscle disorders [9] that contribute to poor balance function [10]. In addition, the combination of frailty and sarcopenia increases the risk of falls [11,12]. In particular, the risk of femoral fracture after a fall is greater in patients with heart failure [13]. Therefore, it is important to focus on improving balance function and decreasing falls in both community-dwelling older adults and those with CVD.

In this context, measures are needed to improve the balance capacity of CVD patients and reduce their risk of falls. Exercise therapy including balance exercises is one effective method of preventing falls [14]. In addition, many CVD patients undergo cardiac rehabilitation (CR); traditionally, CR programs are based on exercise therapy including aerobic exercise and resistance training. However, conventional CR programs do not improve participants’ balance function [15]. As the number of older patients has increased, balance training, flexibility exercises, and secondary preventive interventions have been added to conventional CR programs [16,17]. These modified CR protocols improve not only exercise tolerance but also balance function [18]. For these reasons, we feel that balance training strategies need to be optimized for older adults with CVD.

Several recent studies have shown that exercises using a Balance Exercise Assist Robot (BEAR) improve balance function [19,20,21,22]. Compared with traditional exercises only, those incorporating a BEAR improved dynamic balance among community-dwelling frail and prefrail older adults [19]. In addition, exercises using a BEAR have increased balance function in patients with central nervous system disorders [20] and in older adults with hip fractures [21]. These reports suggest that balance practice involving a BEAR might also improve the balance function of older adults with CVD. Therefore, the aim of the current study was to examine whether adding robotic balance exercises to CR improved the balance ability of older adults with CVD.

## 2. Materials and Methods

### 2.1. Study Population

This prospective interventional study involved patients who were at least 65 years old and who had been hospitalized for worsening CVD (Department of Cardiology, National Center for Geriatrics and Gerontology, Obu, Japan) between August 2019 and December 2021. Participants performed a cardiopulmonary exercise test; underwent laboratory measurements, echocardiography, and a physical function evaluation; and completed questionnaires, including the Fall Efficacy Scale-International (FES-I), Geriatric Depression Scale (GDS), and Mini Nutritional Assessment-Short Form (MNA-SF).

Standard echocardiographic measurements were obtained in accordance with the current guidelines of the European Association of Cardiovascular Imaging [23]. All echocardiographic examinations were performed by 4 senior sonographers who are accredited members of the Japan Society of Ultrasonics in Medicine and registered medical sonographers. Transthoracic echocardiograms were performed in the left lateral decubitus or supine position. All patients underwent M-mode, 2-dimensional, pulsed, and color tissue Doppler echocardiography from a phased-array electronic ultrasound system using Vivid 7 (GE Healthcare, Wauwatosa, WI, USA) or iE33 (Philips Healthcare, Eindhoven, The Netherlands) software. The left ventricular ejection fraction was measured according to the modified Simpson’s method. The peak flow velocities at the mitral level during rapid filling (E) and atrial contraction (A), the E/A ratio, and the deceleration time were calculated from the pulsed Doppler echocardiography data. We recorded the tissue Doppler imaging wave of the mitral annulus from the septal side of the apical 4-chamber view and analyzed the early diastolic filling velocity (E’). The operators making the echocardiographic evaluations were blinded to the patients’ clinical status. These assessments were performed just before discharge, after the patients had been medically stabilized.

The inclusion criteria were structural heart disease consisting of coronary artery disease (having experienced angina pectoris or myocardial infarction, with a history of revascularization procedures); symptomatic heart failure (non-ischemic cardiomyopathy, ischemia, tachycardia, bradycardia, valvular, or hypertension); and others. Non-ischemic cardiomyopathies were defined as ventricular myocardial abnormalities in the absence of coronary artery disease or valvular, pericardial, or congenital heart disease [24]. Tachycardia and bradycardia included atrial, supraventricular, and ventricular arrhythmias; sick sinus syndrome; and atrioventricular block in the absence of structural heart disease. Valvular heart disease was diagnosed on the basis of hemodynamic or echocardiographic findings or a history of valvular or congenital cardiac surgery according to the American College of Cardiology-American Heart Association guideline [25]. Hypertension was defined as a systolic blood pressure ≥140 mmHg, a diastolic blood pressure ≥90 mmHg, or a history of treatment for hypertension. Included as “others” were aortic disease, peripheral artery disease, and other vascular diseases. Worsening heart failure was defined as a clinical syndrome comprising symptoms and/or signs due to structural and/or functional cardiac abnormality and accompanied by elevated natriuretic peptide levels and/or objective evidence of pulmonary or systemic congestion [26].

Exclusion criteria were severe respiratory dysfunction (i.e., patients receiving long-term oxygen therapy for respiratory disease), liver dysfunction (Child–Pugh score class C), stroke, renal dysfunction (reduced glomerular filtration rate and albuminuria category G5), malignant tumors with a prognosis of less than 1 year, criteria corresponding to the CR contraindications in the Guidelines for Rehabilitation in Patients with Cardiovascular Disease of the Japanese Circulation Society [27], cognitive inability to understand how to operate the BEAR, and visual or hearing impairments that could interfere with playing the games.

The study protocol complied with the Declaration of Helsinki, and written informed consent was obtained from each subject. The Ethics Review Board of Nagoya University approved the study (approval no. 2020).

### 2.2. Clinical Characteristics

Frailty was defined according to the revised Japanese version of the Cardiovascular Health Study (J-CHS) criteria [28]. J-CHS assesses 5 components: weight loss, physical activity, tiredness, muscle weakness, and gait speed. Frailty is defined as the presence of signs or symptoms associated with at least 3 of the 5 components, prefrailty as showing signs or symptoms consistent with 1 or 2 components, and robustness as having no features attributable to any of the components.

### 2.3. BEAR

**Figure 1:**
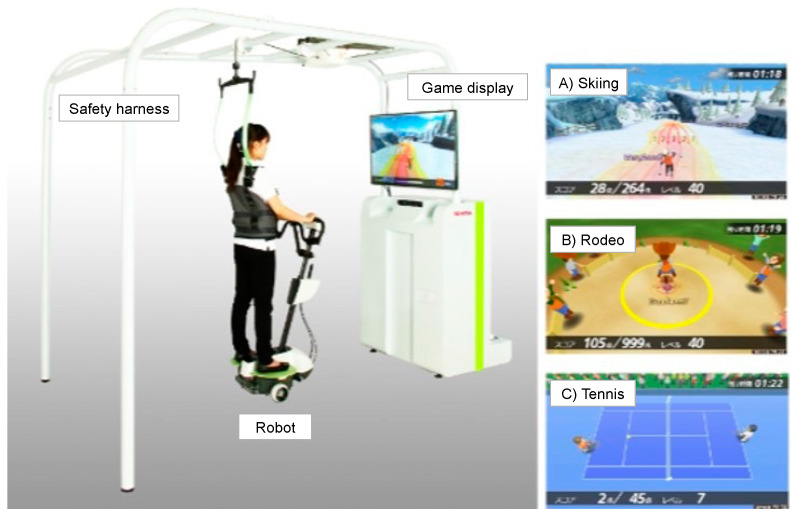
Balance Exercise Assist Robot (BEAR). The BEAR moves backward–forward and left–right according to shifts in the operator’s center of gravity. These movements can be incorporated into balance exercises by playing 3 games: (**A**) a skiing game, which involves using left–right movement; (**B**) a rodeo game, which requires responses to disturbance stimuli from the BEAR; and (**C**) a tennis game, which responds to forward–backward movement.

The BEAR (Toyota Motor Corporation, Aichi, Japan) used in this study had 2 wheels, an in-wheel motor controlled by an inverted pendulum system, and a foot plate on either side (Figure 1 and  Supplementary Video S1). The BEAR moves backward and forward and left and right according to shifts in the operator’s center of gravity. Balance exercise that promotes movement in these 4 directions is achieved by playing 3 games, which focus on different skills: (A) a skiing game, which requires left–right movement; (B) a rodeo game, which requires keeping the robot stationary against irregular disturbances; and (C) a tennis game, which involves forward–backward movement (Figure 1). The level of difficulty was adjusted to suit the individual, and the users performed the repetitive movements automatically [21].

To ensure safety during the exercises, we prepared a 2.4 m × 2.0 m space in the exercise room for participants’ exclusive use. In addition, participants wore a safety harness to limit fall risk; no lifting force was applied to reduce weight-bearing.

### 2.4. CR Program

Participants performed balance exercises by using the BEAR and aerobic exercises by using an ergometer. The intervention was performed on an outpatient basis once weekly for four months, with sixteen sessions in total. Each balance practice session using the BEAR comprised 12 episodes: 4 rounds each of the 3 games, with each round being 1.5 min long. Aerobic exercise was performed for 15 min under loading as determined from the results of cardiopulmonary exercise testing. The load was increased gradually according to the subjective exercise intensity: when the Borg score was less than 12, the load was increased slightly; when the Borg score was 15 or higher, we reduced the number of games played or shortened the duration of aerobic exercise.

### 2.5. Measurements

Peak oxygen uptake (peak VO_2_), 10 m gait test (gait speed), Short Physical Performance Battery (SPPB) score, timed up-and-go (TUG) test, muscle strength of knee extension (knee extension), FES—I, GDS, and MNA—SF were assessed just before discharge after the patients had been medically stabilized and at the end of the 4-month intervention period.

#### 2.5.1. Peak Oxygen Uptake

Each patient underwent cardiopulmonary exercise testing (AE-310S, NIHON KOHDEN, Tokyo, Japan) on a cycle ergometer at a progressively increasing work rate to maximal tolerance. The test protocol was conducted according to the recommendations of the American Thoracic Society and American College of Chest Physicians [29]. Gas-exchange data were obtained breath by breath, and peak VO_2_ was determined as the highest oxygen value recorded during the test.

#### 2.5.2. The 10 m Gait Test

Participants were instructed to walk twice in a 16 m direction at a comfortable pace [30]. The time taken to cover 10 m along the walkway was measured, and the gait speed was calculated from the measured time.

#### 2.5.3. SPPB

The SPPB evaluates lower limb function [31] through its 3 components: balance tests (closed leg standing, semi-tandem standing, tandem standing), walking time, and standing from a seated position. Its reliability, validity, and feasibility in older adults have been reported [32]. The maximum score is 12 points: the higher the score, the better the physical function.

#### 2.5.4. TUG Test

The TUG test measures the time required for a participant to stand up from an armchair, walk 3 m, turn, return to the chair, and sit down again [33].

#### 2.5.5. Muscle Strength of Knee Extension

The strength of knee extension was measured by using a handheld dynamometer (μ-tas F-1; Anima, Tokyo, Japan). Knee extension was tested while the participant was seated, with the knee and hip flexed at 90° [34]. Each strength test was performed twice, and the best result was recorded.

#### 2.5.6. FES-I

The FES-I was developed by the Prevention of Falls Network Europe [35]. FES-I is a 16-question evaluation that assesses anxiety about falling in daily life or social activities. Respondents classify their anxiety about falling as “no concern” (1 point), “somewhat concerned” (2 points), “fairly concerned” (3 points), or “very concerned” (4 points). The points for the 16 questions are summed (16 to 64 points in total): the higher the score, the greater the fear of falling.

#### 2.5.7. GDS

The GDS is a 15-item indicator of depression that is used in Japan [36]. The maximum score is 15 points, and higher scores represent more severe depression. A score of 10 or more indicates the presence of depression.

#### 2.5.8. MNA-SF

The MNA-SF consists of 6 items. MNA-SF considers food intake, weight loss, and physical and psychological stress over the last 3 months, as well as body mass index [37]. MNA-SF scoring identifies 3 nutrition levels: 0 through 7 points, malnourished; 8 through 11 points, at risk of malnutrition; and 12 through 14 points, normal nutrition status.

### 2.6. Statistical Analysis

Continuous variables are reported as means and standard deviations (mean ± SD). Categorical data are reported as the percentage of all subjects. Parameters obtained just before discharge after the patients had been medically stabilized were compared with those at the end of the 4-month intervention period by using paired *t*-tests and Mann–Whitney U-tests. Normality of data distribution was confirmed by using the Shapiro–Wilk test. All statistical analyses were performed by using SPSS version 27.0 (IBM, New York, NY, USA), with significance defined as *p* < 0.05.

## 3. Results

### 3.1. Clinical Characteristics

**Figure 2:**
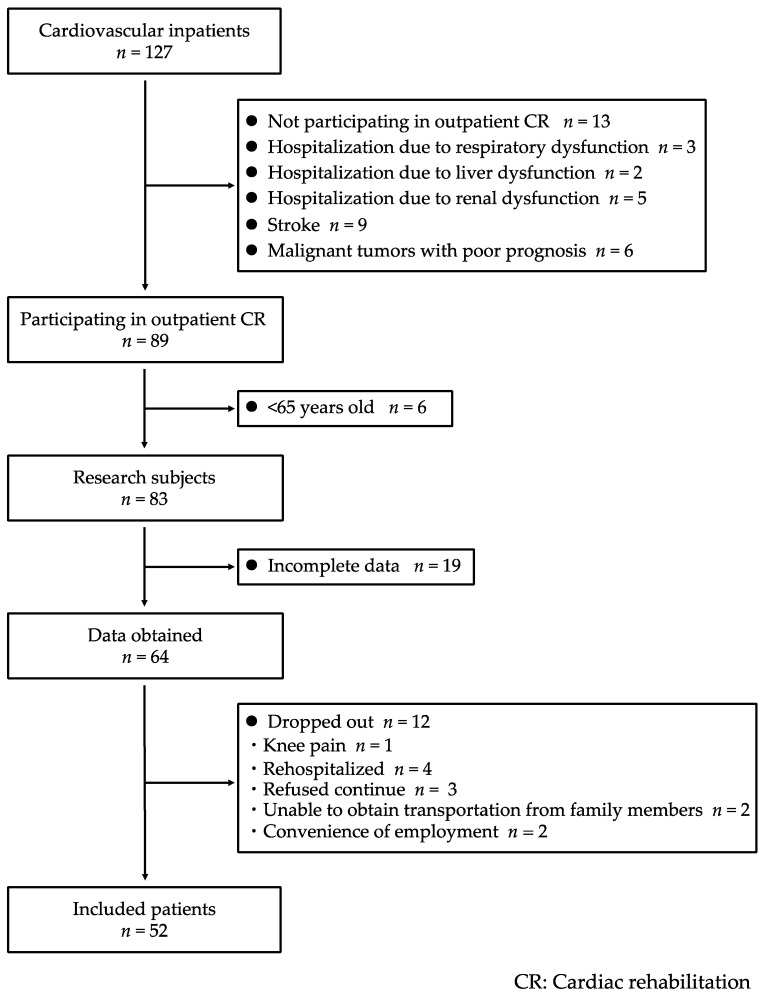
Flow chart of participant inclusion. Of the 127 patients admitted to the cardiology department of our hospital between August 2019 and December 2019, 52 were included in the study. CR, cardiac rehabilitation.

Figure 2 is a flow chart that describes how we derived the study population. The demographic and baseline clinical characteristics of the 52 patients enrolled (Table 1) were obtained once the patients were clinically stable in terms of their CVD. The average age of the study population was 76.9 ± 6.8 years; 53.8% of subjects were male; 88.4% of subjects were patients with worsening heart failure; the brain natriuretic protein concentration was 164.0 ± 190.9 pg/mL; the left ventricular ejection fraction was 57.7% ± 12.0%; and 28.8% of patients were frail. In terms of underlying diseases, 28.8% of participants had ischemic heart disease, and 23.1% had tachycardia-induced heart failure, such as atrial fibrillation or atrial flutter, or an implantable cardioverter defibrillator; 82.7% of subjects had heart failure with a preserved ejection fraction.

### 3.2. Effect of CR including BEAR Exercises

**Figure 3:**
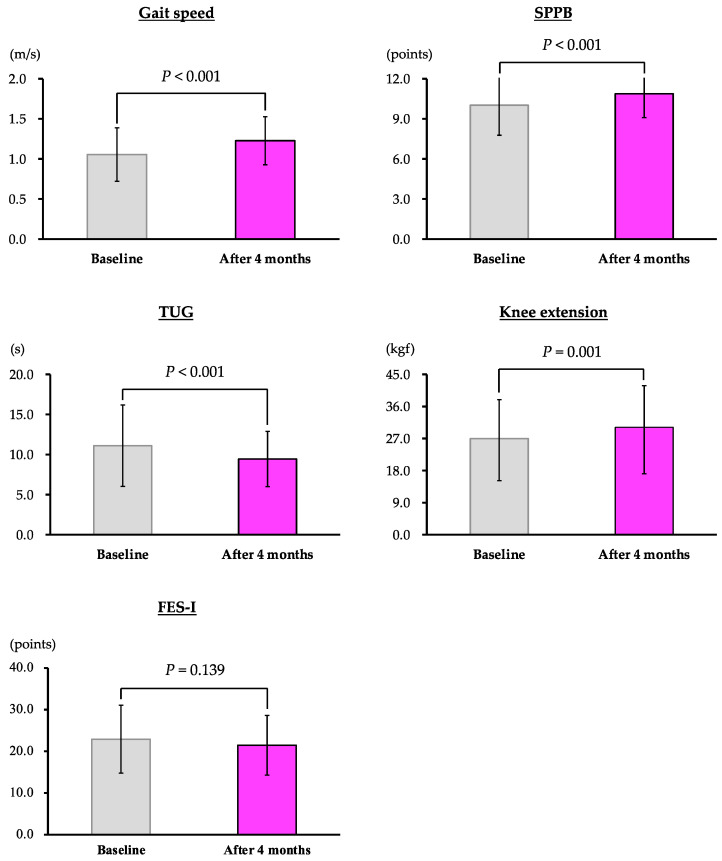
Intervention effect of CR including BEAR exercises (all patients). Changes in 10 m gait test (gait speed), Short Physical Performance Battery score (SPPB), timed up-and-go (TUG) test, muscle strength of knee extension (knee extension), and fear of falling (Fall Efficacy Scale—International, FES-I).

Table 2 shows the results of a comparison of each parameter just before discharge after the patients had been medically stabilized (baseline) and at the end of the 4-month intervention period (after 4 months). Overall, participants achieved significant improvements in gait speed (*p* < 0.001), SPPB score (*p* < 0.001), TUG time (*p* < 0.001), and knee extension (*p* = 0.001; Table 2 and Figure 3) after completing CR including BEAR exercises. In particular, the participants who were prefrail at baseline showed significant improvements in gait speed (*p* < 0.001), TUG time (*p* = 0.008), and knee extension (*p* = 0.003). After completing CR including BEAR exercises, frail participants showed significant improvements in gait speed (*p* < 0.001), SPPB score (*p* = 0.004), TUG time (*p* = 0.004), and knee extension (*p* = 0.042). Robust patients showed no difference in any of the parameters after CR including BEAR exercises. No adverse events occurred.

Table 3 shows the comparison between parameters at baseline and after 4 months of CR including BEAR exercises in male and female participants: gait speed (male; *p* = 0.001, female; *p* < 0.001), SPPB (male; *p* = 0.003, female; *p* = 0.029), TUG (male; *p* = 0.002, female; *p* = 0.010), and knee extension (male; *p* = 0.007, female; *p* = 0.048). Overall, both sexes showed similar results after participating in CR and BEAR exercises for 4 months.

## 4. Discussion

To assess whether adding a recently developed device, the BEAR, to CR improves the balance ability of older adults with CVD, we conducted a prospective study in 52 adults (age ≥65 years) who were admitted to our hospital for worsening CVD. After discharge, participants used a BEAR to perform balance exercises and an ergometer to perform aerobic exercises on an outpatient basis once weekly for four months (i.e., 16 sessions in total). We found that CR intervention involving a BEAR improved gait speed, SPPB score, TUG time, and knee extension—but not peak VO_2_ or FES-I—in older adults with CVD. In addition, no adverse events, such as fatal arrythmia, syncope, worsening heart failure, or acute coronary syndrome, occurred while participants performed BEAR exercises; one participant discontinued BEAR exercises owing to knee pain.

Several robotic assist devices have been shown to be effective components of exercise therapy protocols. Exercise therapy with a robotic walking support device improved the exercise ability and quality of life of patients with heart failure [38]. An exoskeleton-type robot was safe to use in patients with heart failure, and the subjects showed a high interest in using this tool [39]. To our knowledge, our current study is the first to report on exercise therapy using a BEAR in CR protocols for older adults with CVD.

In a previous study, the use of a BEAR improved gait speed, TUG time, and the muscle strength of knee extension among community-dwelling frail and prefrail older adults [19]. We obtained similar results with our current study population. Furthermore, the reported Minimal Clinically Important Difference (MCID) for gait speed is 0.05 m/s [40], for SPPB is 0.5 points [40], for TUG time is 1.2 s [41], and for muscle strength of knee extension is 3.3 kgf [42]. In the current study, improvements in these various physical function parameters after 4 months of CR including BEAR exercises were equivalent, or superior, to the MCID. Additional beneficial features of a BEAR as a mechanism for improving balance function include: (1) high similarity between BEAR game tasks and ankle and hip posture strategies; (2) appropriate and automatic adjustment of game difficulty; and (3) the ability of suitably difficult games to cue an operator to actively move their center of gravity and thus refine posture strategies [19].

Our current study represents a first attempt at incorporating a BEAR into CR intervention for CVD patients, who we thought might benefit by learning posture strategies through BEAR exercises. Previous studies assessing balance training were diverse in terms of exercise type and duration, and the reported effects were likewise varied [43,44]. Therefore, we consider that one advantage of a BEAR is that the difficulty level is tailored automatically and appropriately to each subject, so that essentially everyone can practice balancing correctly [20]. In our sub-analyses, the prefrail and frail groups—but not robust participants—showed significantly improved balance after 4 months. This finding suggests that frail CVD patients might particularly benefit from CR programs that incorporate BEAR exercises to improve balance function.

In several previous studies, CR improved peak VO_2_ in CVD patients [45], as did exercise therapy in frail older adults [46]. However, adding BEAR exercises to CR did not alter peak VO_2_ in our current study, similar to the lack of effect of CR on peak VO_2_ in frail older adults with CVD [47]; the underlying reason remains unknown. In the present study, 92% of participants were prefrail or frail, and whether the degree of frailty or cardiac dysfunction influenced our results is unclear. In addition, age is a reported determinant of improvement in exercise tolerance [48]; the average age of our participants exceeded 75 years and might therefore have influenced our current results. In contrast, after using BEAR for 4 months, participants’ nutritional status (i.e., MNA-SF score) was significantly improved, and their depression (i.e., GDS score) showed a trend toward improvement. These results suggest that, even without increases in peak VO_2_, CR using BEAR might mitigate frailty overall.

In the current study, 88.4% of participants had worsening heart failure, and those with heart failure and a preserved ejection fraction accounted for 83.7%. CR has been reported to improve the prognosis of frail patients with heart failure with preserved ejection fraction [49]. Our data might be generalizable to all cardiovascular system diseases for which CR is appropriate. The success of CR is highly dependent on long-term patient compliance [17], and determining the appropriate exercise intensity is important for the rehabilitation of patients with CVD because it influences compliance as well as the effectiveness and medical safety of the incorporated exercise training [50]. How best to determine the appropriate intensity of both aerobic and resistance exercises for patients with CVD warrants urgent attention. Furthermore, neither the optimal intervention period nor the optimal exercise intensity for exercise therapy involving a BEAR has been determined, and these issues merit further investigation.

Although FES-I scores did not improve after the 4-month BEAR protocol, none of our participants had a fall-induced fracture during the intervention period. The average FES-I score for our participants was 22.91 ± 8.16 points, compared with reported cut-off values of 24 points for community-dwelling elderly patients [51], 23 points for diabetic female patients [52], and 28 points for stroke patients [53]. In addition, the average FES-I score for our study population was lower than that for older adults with hip fractures (32.0 ± 11.3 points) [21]. Compared with their peers, the subjects in our current study may have had less fear of falling, and performing BEAR exercises may have further helped these older adults with CVD avoid fractures due to falls. These ideas should be explored in a future study involving more patients.

Limitations of the current study include its small number of participants (fewer than 50% of patients initially identified were enrolled in the study), the lack of a control group, use of a single facility, and lack of a follow-up survey of cardiovascular events. We considered comparing our current results with data from a historical population that had already performed conventional CR and could serve as a control group, but we were unable to do so because of lack of sufficient data and appropriately similar patients. Owing to the small number of participants, we were unable to verify the effects of age, sex, type or severity of disease, and comorbidities on our findings. Moreover, owing to the lack of a control group, the effect of balance exercises using a BEAR could not be separated from those due to other components of the CR intervention. However, we can attest that using a BEAR is safe in older adults with CVD. In the future, we need to investigate whether CR including BEAR exercises reduces the risks of falls, fractures, readmissions, and early deaths in this population.

## 5. Conclusions

CR including BEAR exercises improved physical functioning—including balance—in older adults with CVD. Despite the small number of subjects, robust subjects showed no changes, whereas those who presented as frail or prefrail improved in a number of parameters. The effects of CR including BEAR exercises on these patients and their prognoses should be verified through randomized controlled trials and follow-up surveys.

## Figures and Tables

**Table 1:** Clinical characteristics of study participants (*n* = 52).

Age (years)	76.9	±	6.8
Sex (male, %)	53.8		
Body mass index (kg/m^2^)	22.7	±	3.3
**Coronary risk factor**			
Diabetes mellitus (*n*, %)	8 (15.4)		
Dyslipidemia (*n*, %)	17 (32.4)		
Tobacco users (*n*, %)	1 (1.9)		
**Underlying diseases**			
Worsening heart failure (*n*, %)	46 (88.4)		
Non-ischemic cardiomyopathy (*n*, %)	2 (3.8)		
Ischemic heart disease (*n*, %)	15 (28.8)		
Tachycardia-induced (*n*, %)	12 (23.1)		
Atrial fibrillation/Atrial flutter (*n*, %)	11 (21.2)		
Implantable cardioverter defibrillator (*n*, %)	1 (1.9)		
Bradycardia; Implanted pacemaker (*n*, %)	5 (7.7)		
Valvular (*n*, %)	4 (7.7)		
Post TAVI (*n*, %)	3 (5.9)		
Post MVR (*n*, %)	1 (1.9)		
Hypertension (*n*, %)	4 (7.7)		
Others (*n*, %)	4 (9.6)		
Aortic disease (*n*, %)	2 (3.8)		
Atrial septal defect (*n*, %)	2 (3.8)		
Post PCI/CABG (*n*, %)	6 (11.6)		
**Medication**			
Diuretics (*n*, %)	20 (38.5)		
Tolvaptan (*n*, %)	8 (15.4)		
ACE-I/ARB (*n*, %)	23 (44.2)		
β blocker (*n*, %)	24 (46.2)		
Spironolactone (*n*, %)	6 (21.1)		
Anticoagulant (*n*, %)	25 (48.1)		
Antiplatelet agent (*n*, %)	25 (48.1)		
**Laboratory data**			
BNP (pg/mL)	164.0	±	190.9
Hemoglobin (mg/dL)	12.8	±	1.7
Total protein (g/dL)	6.8	±	0.6
Albumin (g/dL)	3.9	±	0.5
Total cholesterol (mg/dL)	173.8	±	37.1
eGFR (mL/min/1.73 m^2^)	63.4	±	17.0
**Echocardiography**			
Left atrial dimension (mm)	40.6	±	9.3
LVEF (%)	57.7	±	12.0
< 40% (*n*, %)	5 (9.6)		
41–50% (*n*, %)	4 (7.7)		
E/e′	14.3	±	5.4
**CPX**			
Respiratory exchange ratio	1.2	±	0.1
Maximal workload (W)	58.6	±	26.1
Predicted peak VO_2_ (%)	62.7	±	17.5
**Frailty**			
J-CHS (robust/prefrail/frail) (%)	7.7/63.5/28.8		
**Others**			
Depressive symptoms (*n*, %)	4 (7.7)		
Educational level (years)	11.8	±	2.4

ACE-I/ARB, angiotensin-converting enzyme inhibitor/angiotensin II receptor blocker; BNP, brain natriuretic peptide; CABG, coronary artery bypass graft; E/e′, ratio of transmitral Doppler early filling velocity to tissue Doppler early diastolic mitral annular velocity; eGFR, estimated glomerular filtration rate; J-CHS, Japanese version of the Cardiovascular Health Study criteria; LVEF, left ventricular ejection fraction; MVR, mitral valve reconstruction; PCI, percutaneous coronary intervention; TAVI, transcatheter aortic valve implantation. Depressive symptoms: the number of participants with GDS of 10 points or more where applicable, data are given as means ± SD.

**Table 2:** BEAR effects.

		Baseline				After 4 Months			*p*
**All participants (*n* = 52)**									
	Peak VO_2_ (mL/min/kg)	14.09	±	3.79		14.53	±	3.88	0.222
	Gait speed (m/s)	1.06	±	0.33		1.23	±	0.30	<0.001
	SPPB (points)	10.02	±	2.25		10.88	±	1.79	˂0.001
	TUG (s)	11.11	±	5.07		9.45	±	3.45	˂0.001
	Knee extension (kgf)	26.97	±	11.78		30.13	±	13.04	0.001
	FES-I (points)	22.91	±	8.16		21.45	±	7.17	0.139
	GDS (points)	2.90	±	3.38		2.21	±	2.76	0.057
	MNA-SF (points)	9.48	±	2.37		12.03	±	2.05	<0.001
	LVEF (%)	59.16	±	9.88		58.58	±	9.93	0.366
	E/e’	14.03	±	4.32		13.77	±	4.73	0.638
	BNP (pg/mL)	29.79	±	10.26		26.71	±	9.92	0.264
**Pre-frail participants (*n* = 33)**									
	Peak VO_2_ (mL/min/kg)	15.08	±	3.68		15.59	±	3.86	0.231
	Gait speed (m/s)	1.14	±	0.29		1.29	±	0.29	˂0.001
	SPPB (points)	10.72	±	1.67		11.14	±	1.66	0.090
	TUG (s)	9.54	±	2.86		8.73	±	2.69	0.008
	Knee extension (kgf)	27.89	±	11.88		32.33	±	13.31	0.003
	FES-I (points)	19.56	±	3.97		19.48	±	3.69	0.899
	GDS (points)	1.50	±	1.46		1.27	±	1.20	0.394
	MNA-SF (points)	9.78	±	2.65		11.83	±	2.23	0.005
	LVEF (%)	60.76	±	8.83		60.04	±	7.01	0.412
	E/e’	12.87	±	3.94		12.98	±	3.39	0.839
	BNP (pg/mL)	113.72	±	136.58		142.00	±	170.79	0.254
**Frail participants (*n* = 15)**									
	Peak VO_2_ (mL/min/kg)	12.64	±	3.64		12.49	±	3.37	0.875
	Gait speed (m/s)	0.79	±	0.25		1.05	±	0.27	˂0.001
	SPPB (points)	8.47	±	2.72		10.20	±	2.14	0.004
	TUG (s)	15.09	±	6.80		11.46	±	4.35	0.004
	Knee extension (kgf)	18.73	±	8.29		21.32	±	9.75	0.042
	FES-I (points)	29.79	±	10.26		26.71	±	9.92	0.264
	GDS (points)	6.07	±	4.25		4.33	±	3.92	0.092
	MNA-SF (points)	8.92	±	2.19		12.33	±	1.78	<0.001
	LVEF (%)	54.86	±	12.13		54.12	±	14.34	0.499
	E/e’	17.15	±	3.98		16.05	±	7.21	0.538
	BNP (pg/mL)	284.50	±	259.78		203.50	±	171.94	0.264

BNP, brain natriuretic peptide; E/e’, ratio of transmitral Doppler early filling velocity to tissue Doppler early diastolic mitral annular velocity; FES-I, Fall Efficacy Scale—nternational; GDS, Geriatric Depression Scale; knee extension, muscle strength of knee extension; LVEF, left ventricular ejection fraction; MNA-SF, Mini Nutritional Assessment—Short Form; peak VO_2_, peak oxygen uptake; SPPB, Short Physical Performance Battery; TUG, timed up-and-go. Baseline: just before discharge, after patients had been medically stabilized. After 4 months: 4 months after beginning CR intervention including BEAR exercises.

**Table 3:** BEAR effects in participants according to sex.

	Men (*n* = 28)									Women (*n* = 24)							
	Baseline				After 4 Months			*p*		Baseline				After 4 Months			*p*
Peak VO_2_ (mL/min/kg)	14.46	±	4.14		14.99	±	4.26	0.148		13.63	±	3.34		13.95	±	3.37	0.885
Gait speed (m/s)	1.11	±	0.33		1.25	±	0.28	0.001		1.00	±	0.33		1.20	±	0.32	<0.001
SPPB (points)	10.31	±	1.72		11.08	±	1.83	0.003		9.70	±	2.74		10.65	±	1.75	0.029
TUG (s)	9.98	±	4.30		8.76	±	3.07	0.002		12.39	±	5.64		10.23	±	3.74	0.010
Knee extension (kgf)	33.96	±	11.20		37.52	±	12.43	0.007		19.33	±	6.75		21.74	±	7.55	0.048
FES-I (points)	22.00	±	6.78		20.46	±	5.52	0.141		24.00	±	9.64		22.65	±	8.76	0.456
GDS (points)	2.36	±	2.86		1.68	±	1.68	0.263		3.48	±	3.85		2.78	±	3.54	0.073
MNA-SF (points)	10.18	±	1.98		12.00	±	2.06	0.012		8.75	±	2.59		12.06	±	2.11	<0.001
LVEF (%)	56.37	±	11.36		55.24	±	10.71	0.230		62.74	±	6.13		62.87	±	6.95	0.885
E/e’	12.90	±	4.19		12.73	±	3.99	0.813		15.67	±	4.06		15.28	±	5.39	0.662
BNP (pg/mL)	156.69	±	216.24		164.19	±	199.62	0.814		176.52	±	172.54		137.33	±	119.32	0.214

All abbreviations are as in Table 2.

## Data Availability

Not applicable.

## Supplementary Materials

The following supporting information can be downloaded at: https://susy.mdpi.com/user/manuscripts/displayFile/3b55ff4c75f73a324f46dcd5b5b30a9f/supplementary (accessed on 17 April 2022).
